# A Fluid Mechanical Interpretation of Hysteresis in Rhinomanometry

**DOI:** 10.5402/2011/126520

**Published:** 2011-09-21

**Authors:** T. F. Groß, F. Peters

**Affiliations:** Lehrstuhl für Strömungsmechanik, Ruhr-Universität Bochum, 44780 Bochum, Germany

## Abstract

A hysteresis effect in the pressure/flow rate relationship of nasal breathing has frequently been observed in clinical tests and in lab investigations. Explanations that have been given in the literature are missing a fluid mechanic storage effect coming into play in reciprocating flows. This effect depends primarily on the way the rhinomanometric measurements are set up and not so much on the nose flow itself. This is to be shown by calculations and experiments. The experiments are carried out with orifices because they can represent nose flow and are often implemented in rhinomanometric equipment as flow gauges. To mimic reality also a 1 : 1 nose model is used. It is shown where the hysteresis comes from and what the key parameters for its prediction are. With these results hysteresis in nasal breathing appears in a new light.

## 1. Introduction

The assessment of nasal breathing is facing an extremely convoluted 3D geometry of small and narrow channels in the nasal cavity. These channels are hardly accessible to probe measurements with the additional complication that the internal walls are not rigid. In fact, they are subject to mucosa swelling and even distensible in the region of the nasal vestibule. Therefore, characterization of the flow has to resort to integral measurements which are taken across the entire cavity. Preferably the pressure drop is recorded versus the flow rate. The field of rhinomanometry has been established around this topic. Numerous articles have appeared dealing with in vivo as well as model tests. An account of the state of the art was given by Clement and Gordts in a consensus report [1].

Hysteresis in this context means that the pressure drop across the nasal cavity measured during, for example, the inspiration phase is associated with two different flow rates, one in the increasing and one in the decreasing section of inspiration. The effect has been observed early in connection with lung volume and dead space [2]. Schumacher et al. made an early approach to introduce computer-based data processing into rhinomanometry [3]. They make mention of the word hysteresis and show it in their graphs yet do not dig any deeper. The first paper that sets the focus on hysteresis is by Shi et al. [4]. They provide data along with comprehensive description and analysis. Their conclusion is that hysteresis is almost entirely due to the compliancy of the vestibule section. Fodil et al. suspect that hysteresis depends on breathing level and the kind of disease [5].

The most recent work on the phenomenon was published by Vogt et al. [6]. It includes technological as well as clinical aspects setting standards for measurements and diagnostic interpretation in the so-called 4-phase rhinomanometry. Part of this work appeared in the already mentioned report by Clement and Gordts [1] where it was critically commented as to the possible origin of hysteresis.

Wherever hysteresis has been tackled the following explanations are suggested and considered. 

### 1.1. Inertia

A recurring argument is inertia. The pressure drop involved in driving a flow is needed in part to overcome the dissipative losses and in part to accelerate the flow. Acceleration must be subdivided into changes of velocity in space at fixed time and those in time at fixed location. When inertia is addressed in the present context the time-dependant one is meant. A simple estimation at the end of the next section shows that this contribution is negligible. We refer to normal quiet breathing below 15 cycles/min. For unnatural frequencies up to 88 cycles/min effects of inertia have been shown to exist [7].

### 1.2. Variable Resistance

When inertia is excluded the pressure drop results from dissipation due to the fluid viscosity and velocity changes (direction and value) along the nasal passageway. For a fixed geometry a unique relationship between pressure drop and flow rate exists. Thus, the pressure drop history may be affected when the geometry changes within a breathing cycle. The change of geometry has two sources. One is the periodical nasal flaring that continuously reshapes the vestibule with its valve mechanism [4]. The other is the mucosal swelling. The first is certain to occur (besides the intentional nonperiodic flaring) and play a role while the swelling is unlikely to occur periodically within a cycle. Either effect cannot appear in a solid nose model but may be interlaced with the hysteresis phenomena observed for a real nose.

### 1.3. Changes in Flow Regime

It has been argued that the flow may switch between laminar and turbulent within a half-cycle [4]. This is most likely true because the flow reciprocates between standstill and maximum. However, in order to cause hysteresis it would mean that two different flow states would exist at one pressure drop. For example during expiration at some intermediate pressure drop the flow rate would be smaller in the accelerating part than in the decelerating part. Fluid mechanically this is very unlikely.

This study is to show that the main reason for the observed hysteresis is not to be sought among the above itemized arguments. In fact it is not the nose flow itself that causes hysteresis. It is a storage effect due to compressibility that becomes important in reciprocating flows even if pressure and density changes are small.

We have dealt with the general implications of the storage effect from the fluid mechanics perspective in [8]. In this study we focus on conditions and circumstances familiar in rhinomanometry. One of the severest disadvantages of real nose flow studies is that no reference is available that allows reproducible results. This is why we substitute the nose by an orifice or a nose model that allows reproduction and a sound analysis of the hysteresis effect.

The study begins with the equation of conservation of mass that covers the key issues.

## 2. Materials and Methods

**Figure 1:**
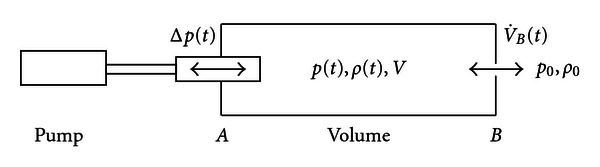
Flow model.

We consider a flow model consisting of a volume *V*, a substitute at *A* for the nose, and an orifice at *B* as sketched in Figure 1.

The task of the orifice at *B* is to measure the flow rate ${\dot{V}}_{B}$ (which is common practice in rhinomanometry). The volume *V* between *A* and *B* accounts for whatever volume there is between nose and orifice in terms of mask, adapters, and hoses which can easily amount to several liters. The nose substitute stands for the pressure drag of the real nose. In rhinomanometry the measured flow rate ${\dot{V}}_{B}\;$at *B* is plotted versus the pressure drop Δ*p* at *A* or vice versa. This is the representation that reveals hysteresis.

Let us think of a given reciprocating flow for example at *A*. Supposed that no air can be stored in *V*, then this flow would appear at *B* in true phase and amplitude. However, air is compressible. Thus air can be stored in *V* at increasing pressure and released at decreasing pressure. Due to the involved low level of pressure changes (a few hundred Pascal) one is readily inclined to neglect this effect. Actually, density changes are small. However, it is not the density change itself that counts. It is rather the derivation of the density with respect to time. And this can be considerable as the equation of conservation of mass is ready to show.

The difference between the mass fluxes at *A* and *B*, ${\dot{m}}_{A}\;$and ${\dot{m}}_{B}$, respectively, is expressed as a storage term on the right side of the equation

(1) $${\dot{m}}_{A}-{\dot{m}}_{B}=V\rho_{0}\frac{d\left(\rho /\rho_{0}\right)}{dt}.$$

Here *ρ*_0_ is the atmospheric reference density downstream of *B*. In general the density ratio *ρ*/*ρ*_0_ depends on pressure and temperature. Yet, with the occurring relatively small variation it is a good approximation to assume isentropic conditions [9]. Then density and pressure are simply related by

(2) $$\frac{\rho }{\rho_{0}}={\left(\frac{p}{p_{0}}\right)}^{1/\kappa },$$

where *κ* names the ratio of specific heats (1.4 for air). Replacing the density in (1) by the pressure leads to

(3) $${\dot{m}}_{A}-{\dot{m}}_{B}=V\rho_{0\;}\frac{1}{\kappa \;}{\left(\frac{p}{p_{0}}\right)}^{\left(1-\kappa \right)/\kappa }\;\frac{d\left(p/p_{0}\right)}{dt}.$$

At this point two approximations are justifiable. The mass fluxes are converted to volume fluxes by division with the mean density of the cycle which is *ρ*_0_. And, being close to unity, the pressure ratio to the power (1 − *κ*)/*κ* is set to one. Then, (3) is well approximated by

(4) $${\dot{V}}_{A}-{\dot{V}}_{B}=\frac{V}{\kappa }\frac{d\left(p/p_{0}\right)}{dt}.$$

In [8] it was shown that the orifice can be used in the involved reciprocating flows and that the flow rate is obtained from

(5) $${\dot{V}}_{B}=A_{B}\sqrt{\frac{2\left(p-p_{0}\right)}{\zeta_{B}\rho_{0}}}.$$

Here *A*_*B*_ and *ζ*_*B*_ are the area of the bore and the loss coefficient of the orifice, respectively. We use (5) in (4) and normalize the time with the cycle period *T*, that is, *τ* = *t*/*T*. Then, (4) yields

(6) $${\dot{V}}_{A}=A_{B}\sqrt{\frac{2\left(p-p_{0}\right)}{\zeta_{B}\rho_{0}}}+\frac{V}{\kappa T}\frac{d\left(p/p_{0}\right)}{d\tau }.$$

The flow rate at *A* depends on the substitute used. However, whatever is actually used ${\dot{V}}_{A}$ will be a function of the pressure loss Δ*p* across the substitute (or nose), that is, ${\dot{V}}_{A}={\dot{V}}_{A}\left(\Delta p\right)$. This means that Δ*p* and the pressure across the orifice at *B*, being equivalent to ${\dot{V}}_{B}$ through (5), are directly related. Note that the trivial case ${\dot{V}}_{A}={\dot{V}}_{B}$ emerges only when the storage term becomes negligible.

In order to grasp the impact of the storage term the nose may be substituted by an orifice itself. Then, we have

(7) $$A_{A}\sqrt{\frac{2\Delta p}{\zeta_{A}\rho_{0}}}=A_{B}\sqrt{\frac{2\left(p-p_{0}\right)}{\zeta_{B}\rho_{0}}}+\frac{V}{\kappa T\;}\frac{d\left(p/p_{0}\right)}{d\tau }.$$

This equation allows us to carry out an exemplary calculation if we formally assume a sinusoidal flow rate at *B* with the maximum ${\dot{V}}_{Bm}$

(8) $${\dot{V}}_{B}={\dot{V}}_{Bm}{\sin\left(2\pi \tau \right)=A}_{B}\sqrt{\frac{2\left(p-p_{0}\right)}{\zeta_{B}\rho_{0}}}.$$

The ratio  *p*_0_/*ρ*_0_ is replaced by the equation of state and the speed of sound [9] *a*_0_^2^ = *κR*_*s*_ *T*_0_ at atmospheric temperature *T*_0_:

(9) $$\frac{p_{0}}{\rho_{0}}=R_{s}T_{0}=\frac{a_{0}^{2}}{\kappa }.$$

Then, we get from (8) for the pressure ratio and its derivative

(10) $$\frac{p}{p_{0}}=\frac{\kappa \zeta_{B}{\dot{V}}_{Bm}^{2}}{2{\left(a_{0}A_{B}\right)}^{2}}\sin^{2}\left(2\pi \tau \right)+1,$$

(11) $$\frac{d}{d\tau }\left(\frac{p}{p_{0}}\right)=\frac{2\pi \kappa \zeta_{B}{\dot{V}}_{\mathrm{Bm}}^{2}}{{\left(a_{0}A_{B}\right)}^{2}}\sin\left(2\pi \tau \right)\cos\left(2\pi \tau \right).$$

Inserted into (7) yields after rearrangement

(12) $$\frac{\Delta p}{p_{0}}=\frac{\kappa \zeta_{A}}{2}{\left\{\frac{{\dot{V}}_{Bm}^{}\sin\left(2\pi \tau \right)}{a_{0}A_{A}}\right\}}^{2}{\left[1+\frac{2\pi \zeta_{B}V{\dot{V}}_{Bm}^{}}{{\left(a_{0}A_{B}\right)}^{2}T}\cos\left(2\pi \tau \right)\right]}^{2}.$$

**Figure 2:**
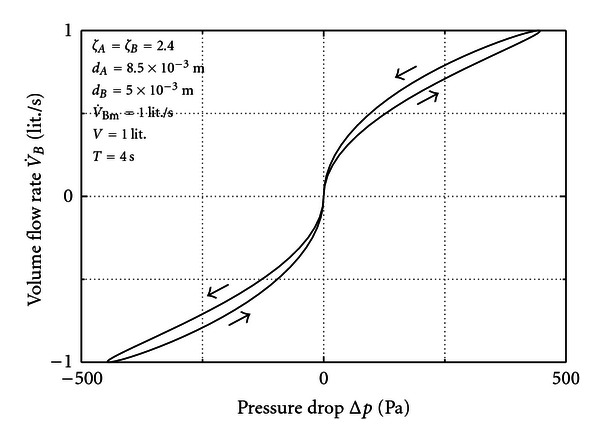
Hysteresis calculated from (12).

An example calculated from this equation is shown in Figure 2. The involved parameters are in the scope of nose flow. The areas *A*_*A*_ and *A*_*B*_ are represented by the diameters *d*_*A*_ and *d*_*B*_.

A pronounced hysteresis emerges, the arrows indicating the path the loop is run through. It is the greatest where the storage term of (11) has its maximum. It vanishes at the turning point between in- and expiration where flow rate and pressure approach zero. Although written for a special case (11) and (12) demonstrate the principle influence of the various parameters via the term in square brackets that represents the storage effect. Predominantly, when *V* goes to zero or *T* gets very large the effect diminishes. Enhancing contributions come also from the loss coefficients and the maximum flow rate. Other than this the bore areas of the orifices have an attenuating effect. The reference pressure *p*_0_ and speed of sound *a*_0_ are basically invariable in this problem.

In this example pressure and flow rate collapse at the zero point with no time shift. The reason is the sinus at *B*, which results in a sinusoidal function on the right side of (12). When the sinus is zero, then the right side is zero as is the left side. This shows that according to the type of function at *B* or *A* the result may be different and a hysteresis at the zero point can appear. We have shown in [8] that this can happen under relatively extreme conditions.

At this point it is convenient to give an estimation of the inertia term referred to in the introduction. With (8) the maximum acceleration occurs where the cosine is one, that is, 2*πw*_max⁡_. Here *w*_max⁡_ is the maximum velocity obtained from ${\dot{V}}_{Bm}/A$. The force due to the maximum acceleration is then the density times the volume of a typical cavity (length × area *A*) times the acceleration. This force is equilibrated by a pressure times the area. We get the order of 1 Pa based on an area of 300 mm², a length of 50 mm, and a density of 1,2 kg/m³. From Figure 2 we see that this contribution to the total pressure is in fact very small as stated in the introduction.

## 3. Experimental Results

### 3.1. Nose Represented by an Orifice

A crank-driven piston pump as described in [8] was employed to generate a reciprocating flow through two orifices placed at *A* and *B* with a volume *V* in between. The pressure measurements were conducted with very accurate Baratrons. Measured is the flow rate at *B* and the corresponding pressure drop at *A*. 

**Figure 3:**
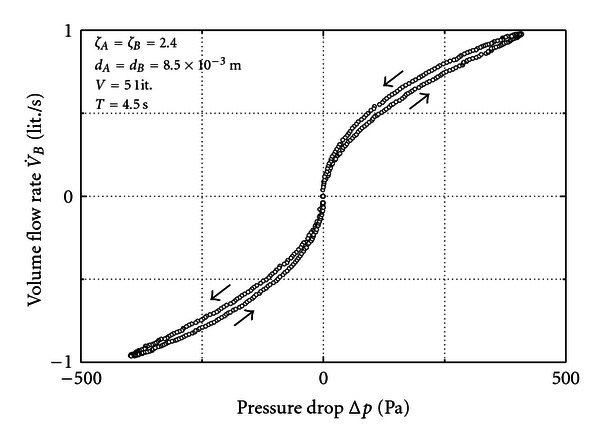
Measurement for two equal orifices.

Figure 3 presents a result measured for the parameters inserted in the figure. The two orifices are identical and the volume is 5 liters. The loop is run through as the arrows indicate. A clear hysteresis is observed.

Given the situation of Figure 3 it was found that the hysteresis becomes imperceptible when the volume reduces to the order of one liter.

Note that Figure 3 resembles Figure 2 although the parameters are different. The reason is that a small volume combined with the small orifice renders roughly the same storage effect as a large volume combined with the large orifice. Despite this similarity a direct comparison is not allowed because in Figure 2 a pure sinus at *B* was supposed which is not perfectly attained by the pump in combination with *V*.

**Figure 4:**
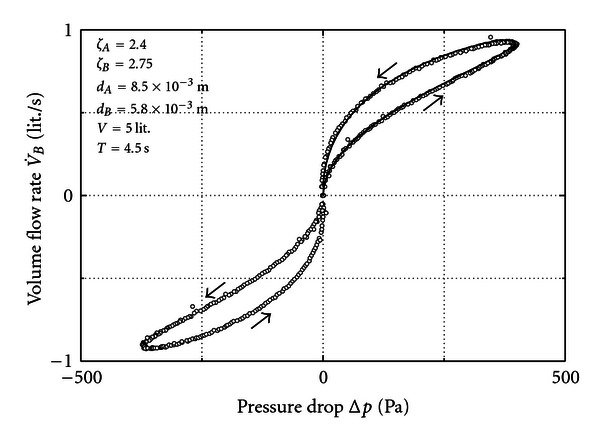
Measurement for unequal orifices (circles) and calculation (line) from (7).

In Figure 4 we leave the volume at 5 liters and reduce the diameter at *B* to 5.8 mm. Then, the result is a strong enhancement of the hysteresis. This includes a reduction of the maximum flow rate although the pump action is the same. 

In the theoretical exemplary case a sinusoidal flow rate at *B* was supposed to ease the analytical calculation of the hysteresis by means of (12). 

In a real case the flow rate at *B* cannot be modeled as a perfect sinus. However, it has a reciprocating character and it is available as a measured signal. Then, in order to calculate the pressure loss at *A* from the flow rate at *B* (7) has to be integrated numerically. In the present experiment with two orifices the piston pump provides a fair sinus but with the volume between pump and *A* the signal at *A* is already distorted. Therefore, even in the present experiment we have to apply (7) for a numerical result of the pressure drop at *A*. This was done exemplarily for one branch of the cycle in Figure 4. The agreement between measured and calculated hysteresis proves that the interpretation based on mass conservation and isentropic change of state is successful.

**Figure 5:**
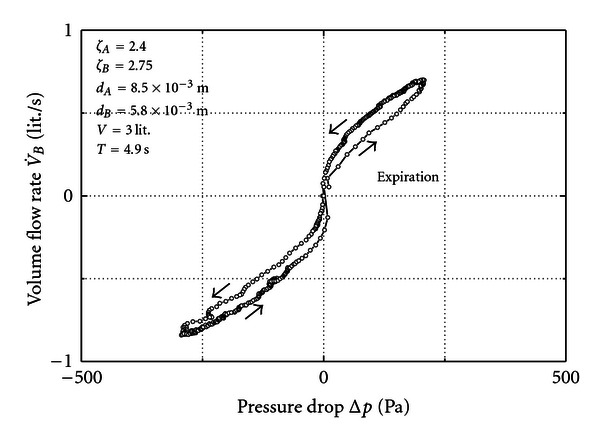
Test person breathing through orifice at *A*.

Finally, to get somewhat closer to a realistic case the piston pump was simply replaced by a test person. By means of a mouth piece the test person was connected to orifice *A* and asked to breathe through the mouth quietly as if breathing through the nose.  Figure 5 provides an example of a full cycle. Evidently the piston pump simulates real breathing quite well except that the real curve exhibits some natural wiggle. In real breathing inspiration and expiration are not necessarily symmetric. Here, expiration is slower than inspiration such that the maximum attained expiration flow rate is smaller than the inspiration one.

The three examples with orifices refer to a limited scope of parameters. Further parameter variations could be pursued to round off the picture of what the effects are quantitatively. Qualitatively (12) informs on the influence of the involved parameters. 

### 3.2. Nose Represented by a Solid Model

**Figure 6:**
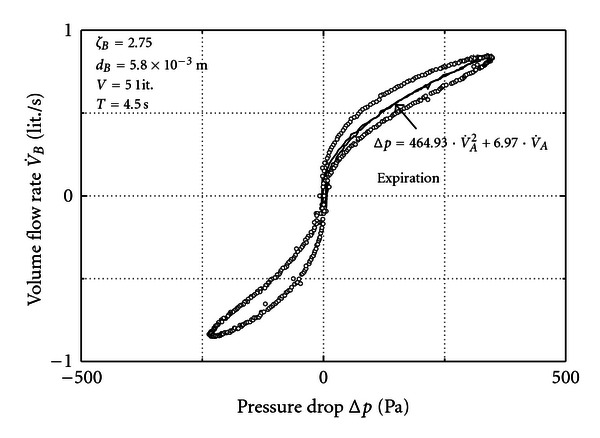
Measurement with a nose model (circles) and recalculation of ${\dot{V}}_{A}$ (line) from (6).

The orifice at *A* is substituted by a 1 : 1 “rapid prototyping” model of the nasal cavity. Otherwise the measuring setup remains unchanged with the piston pump at the *A* side and the orifice at *B* to measure the flow rate. Results appear in Figure 6.

The first observation is that expiration attains much larger pressure than inspiration for equal piston strokes. The only possible explanation is that the flow resistance for expiration is larger than that for inspiration. This property is common with real noses. (Note that the unsymmetry in Figure 5 resulted from different breathing at equal resistances.) The special objective of this plot is to show that the hysteresis can be corrected for. Plotted is the flow rate at *B* versus the pressure drop at *A*, as usual. The effect of the storage term is that ${\dot{V}}_{B}\;$is different from ${\dot{V}}_{A}$. If we want the true ${\dot{V}}_{A}$ we have to calculate the storage term and add it to ${\dot{V}}_{B}\;$as prescribed by (6). The result is shown for expiration as the solid line. As expected the hysteresis of the pressure flow relationship (at *A*) disappears. 

## 4. Discussion

Our theoretical studies show that the equation of mass conservation is in charge of explaining the hysteresis. The fundamental mechanism is the compressibility, more precisely the time derivative of the density. The equations identify the crucial parameters and their influence on the phenomenon. An exemplary calculation based on a sinusoidal flow rate serves to produce an analytical hysteresis curve.

Results on flow rate and pressure measurements are provided. These measurements are based on an orifice substituting the nose and a 1 : 1 model of a nasal cavity. Flow generation is either by a piston pump or by breathing. It is demonstrated how the volume, the flow resistance, and the flow generation affect the shape and the magnitude of the hysteresis. It becomes clear that the hysteresis can be kept small by tuning the involved parameters. It is also shown that a given hysteresis can be recalculated by applying the provided equations. On the other hand a hysteresis recorded for a nose model can be corrected numerically such that the model appears free of hysteresis.

## 5. Conclusion

This study suggests a new interpretation of the hysteresis observed in rhinomanometry. Our claim is that the hysteresis is not inherent to nose flow but to the measuring technique. Whenever the flow rate is measured remote from the nose a storage effect arises that distorts the allocation of flow rate and pressure loss disclosed as hysteresis.

The paper presents calculations and measurements with orifices and a nose model to substantiate the interpretation. The involved quantities are kept in a range relevant to nose flow. The results give a coherent picture leaving no doubt as to the physical reality of the phenomenon.

For future real nose breathing studies we think it mandatory to take the storage effect into account. It may then be even necessary to include further effects. For example, the mask applied to cover the nose may be dilatable. This could enhance the storage effect substantially. Other examples are built-in components like filters or flow sensors because they act like additional flow resistors.

In principle, when the main parameters of influence are accounted for a correction for the hysteresis is conceivable. A severe difficulty which remains is seen in the fact that the shape of the flow rate curve produced by the lungs may vary from stroke to stroke. This would mean that each stroke would need individual correction.

A pragmatic recommendation for the improvement of rhinomanometric measurements with respect to hysteresis is to work on the involved parameters in order to keep the storage term small. To this end the above equations provide a guideline.

## List of Symbols

*V*: Volume (m³)
*p*: Pressure (N/m²)
*ρ*: Density (kg/m³)
$\dot{m}$: Mass flow rate (kg/s)
$\dot{V}$: Volume flow rate (m³/s)
*κ*: Ratio of specific heats
*A*: Bore area (m²)
*d*: Bore diameter (m)
*ζ*: Loss coefficient
*T*: Periodic time (s)
*t*: Time (s)
*τ*: Normalized time
Δ*p*: Difference pressure (N/m²)
*R*_*s*_: Specific gas constant (Nm/(kg K))
*T*_0_: Ambient air temperature (K)
*a*_0_: Speed of sound (m/s)
*a*, *b*: Coefficients.
